# Beliefs about Volunteerism, Volunteering Intention, Volunteering Behavior, and Purpose in Life among Chinese Adolescents in Hong Kong

**DOI:** 10.1100/tsw.2009.32

**Published:** 2009-09-01

**Authors:** Ben M. F. Law, Daniel T. L. Shek

**Affiliations:** ^1^ Department of Social Work, Chinese University of Hong Kong, Hong Kong, China; ^2^ Department of Applied Social Sciences, Hong Kong Polytechnic University, Hong Kong, China; ^3^ Department of Sociology, East China Normal University, Shanghai, China; ^4^ Kiang Wu Nursing College of Macau, China

**Keywords:** Chinese adolescents, volunteering, purpose in life, psychological well-being

## Abstract

The relationships among beliefs about volunteerism, volunteering intention, volunteering behavior, and purpose in life were examined in this study. A total of 5,946 participants completed a series of scales, including the Revised Personal Functions of Volunteerism Scale, Volunteering Intention Scale, and Purpose in Life Scale. The results showed that participants whose purpose in life had different levels also had varied prosocial beliefs about volunteerism, volunteering intention, and volunteering behavior. Purpose in life was associated more strongly with prosocial value function than with other types of beliefs (except understanding function). When different beliefs are grouped, the correlation between purpose in life and other-serving beliefs was higher than that between purpose in life and self-serving beliefs. Purpose in life was also associated with volunteering intention and behavior. Path analyses showed that purpose in life predicted volunteering behavior via beliefs and intention. While other-serving beliefs predicted volunteering behavior directly, self-serving beliefs did not have such direct effect.

### INTRODUCTION

This study aims to explore the relationship between purpose in life and volunteerism among Chinese adolescents in Hong Kong. Human beings have shown enthusiasm in relation to acts that allow them to pursue their purpose in life. In fact, the *New York Times* and *Wall Street Journal* reported huge sales figures posted for Rick Warren's best-selling book *The Purpose Driven Life*[1]. This idea has also been explored in previous literary works. For example, Ralph Waldo Emerson stated once that, "The purpose of life is not to be happy. It is to be useful, to be honorable, to be compassionate, to have it make some difference that you have lived and lived well." Human beings want to approach life with a sense of coherence (sense of order, reason for existence, and understanding) and purpose in life (mission in life, direction, goal orientation)[2,3]. Accordingly, people with a higher level of purpose in life tend to have a sense of direction, mission, and coherence in their daily lives. Purpose in life is thus an indicator of quality of life. In conventional usage, "meaning" refers to sense and coherence, whereas "purpose" refers to intentions, aims, and functions. However, in reference to the inherent ontological significance of life, the terms "purpose in life", "life meaning", and "meaning of life" are often used interchangeably in the field of existential psychotherapy[2]. These terms will all be used in this paper.

The quest to seek one's purpose in life is a universal phenomenon manifested across the life span continuum, cutting across cultures and age groups, especially among the youth. Human history has repeatedly shown us how young people strived for purpose in life. For instance, young people in Nazi Germany believed that building an ethnically superior nation was their life purpose before World War II, whereas young people in China in the 1960s wanted to develop a Communist utopia during the Cultural Revolution[4].

We can understand the importance of purpose in life during adolescence in terms of developmental and existential perspectives. According to different developmental perspectives, adolescence is a crucial time for cultivating a commitment to positive purpose in life. It is marked by enhanced self-understanding and comprehension of one's place in human society and purpose in life. Adolescents struggle to define their identities. Kroger[5] argued that they form their identities by synthesizing earlier identifications into "a new psychological structure, greater than the sum of its parts (p. 3)." By injecting the sense of self, adolescents actively explore their life meanings. In addition, purpose in life is argued to be one of the five types of competencies under self-control in adolescent personality development[6]. At the same time, purpose in life is an important component of spiritual development in adolescence[7].

From the existential perspective, Frankl conceived of life meaning as a process of discovery within a world that is intrinsically meaningful[8]. The prolonged absence of meaning and purpose creates a condition known as existential vacuum. If the will to meaning is not fulfilled, symptoms will rush in to fill the vacuum. These include boredom, apathy, and emptiness. This condition is called noogenic neurosis. Although Frankl's theory is not specifically formulated for adolescents, it is predicted that when an adolescent is aware of his life's meaning, he becomes aware of his own existence and responsibility in the world.

Despite the importance of purpose in life, we do not fully understand its role in adolescent development among Chinese due, perhaps, to several knowledge gaps in the existing literature. First, only a few studies focused on adolescents' purpose in life and it is a neglected area in adolescent research[7]. Second, many studies focused on adolescents' negative behavior and psychopathology[9], and only a few dealt with positive behavior and purpose in life[10]. Third, only a few studies examined the related issues for Chinese adolescents[11].

There are theories that suggest that purpose in life is associated with prosocial behavior. According to Frankl, purpose in life can essentially be attained through three means: productive or creative activities, positive human experiences, and through the experience of unavoidable and negative conditions[8]. Yalom indicated that purpose in life is associated with religious beliefs, self-transcendent values, association with groups, dedication to some social causes, and adoption of clear life goals; purpose in life is associated negatively with self-serving motives[3]. Keyes and Ryff suggested that psychologically healthy people have goals that make their lives meaningful[12]. One characteristic of a meaningful life is that those people tend to be concerned about other people's needs. Prosocial behavior can enhance one's well-being, thus improving one's quality of life as well. Emmons suggested 13 categories of human concerns linked to purpose in life, such as "achievement", "intimacy", and "personal growth and health". One kind of ultimate human concern that is associated with purpose in life is interpersonal concerns[13].

Reker and Wong defined the degree of self-transcendence as an indication of purpose in life[2]. They then proposed four levels of depth regarding the experiences of life meaning. Level 1 is self-preoccupation with hedonistic pleasure and comfort, Level 2 is devotion of time and energy to the realization of personal potential, Level 3 is service to others and commitment to a larger societal or political cause, and Level 4 is transcendence of cosmic meaning and ultimate purpose. According to this framework, people with a higher level of purpose in life tend to focus on transcendental values and the needs of other people rather than self-serving needs. As purpose in life is closely related to transcendental values, it is expected that people with a higher level of purpose in life would be less likely to endorse material and/or self-serving motives.

A review of the literature would demonstrate that purpose in life was indeed positively related to prosocial behavior among adolescents. Shek, Ma, and Cheung showed that purpose in life was positively related to prosocial behavior[10], while Shek, Siu, and Lee showed that purpose in life was positively related to identification with prosocial norms and prosocial involvement[14]. Unfortunately, despite these attempts to examine the relationship between purpose in life and prosocial behavior, no systematic attempt has been made to examine the relationship between purpose in life and adolescent volunteerism.

Volunteering behavior is a special form of prosocial behavior. Unlike spontaneous helping, volunteering behavior is a planned action via the mediation of an organizing agency[15]. There are not many studies about adolescent volunteerism and purpose in life[10,16]. Other studies either do not focus on adolescents[17] or the relationship is about other measures of well-being instead of purpose in life[18]. In addition, most studies on adolescent volunteerism are focused solely on volunteering behavior[19,20]. Cognitive-motivational perspective suggests that one's behavior is influenced both by beliefs and intention[21]. Given that volunteerism is also a type of planned behavior, the roles of beliefs and intention are more prominent.

Among various approaches to understanding beliefs related to volunteerism, the functional approach is the most widely studied[22]. Different people perceive volunteer service with different underlying functions. Some view services as a chance to learn and some set out to serve people in need. The beliefs held for an activity can be the motivating factor for engaging in that particular activity. The functional approach conceptualizes each category of beliefs as a discrete function for the person. Borrowing the ideas of Volunteer Functions Inventory by Clary et al.[22], Law has developed five types of beliefs about volunteerism among adolescents[15]. These beliefs can be further classified as "other-serving beliefs" and "self-serving beliefs" as discussed below.

1. **Prosocial value function (other-serving belief) —** This function primarily refers to serving other people. A prosocial value is the belief that interaction with others and the act of considering other people's interests is more important than self-interests. Adolescents perceive that volunteer service can actualize and express their prosocial values related to altruistic and humanitarian concerns for other members of society. As prosocial value function is the only other-serving belief in this study, the terms "other-serving beliefs", "prosocial beliefs", and "prosocial value function" will be used interchangeably in this paper.
2. **Understanding function (self-serving belief) —** This function refers to the possibility of learning while in service. Adolescents perceive that they can improve their interpersonal skills, acquire skills and knowledge that would help them to serve recipients, and gain a more comprehensive understanding of social issues while serving as volunteers.
3. **Socializing function (self-serving belief) —** This function refers to the socializing aspect of services. Many volunteer services are delivered in groups rather than individuals. In relation to this, adolescents perceive that they can make or meet friends during the service.
4. **Future plan function (self-serving belief) —** This function refers to potential tangible benefits to adolescents. Adolescents regard volunteering as an opportunity to explore future study or career plans. For instance, adolescents in Hong Kong are aware that volunteer service is a definite advantage when applying for admission to better schools or universities.
5. **Well-being function (self-serving belief) —** This function is related to psychological well-being. Adolescents regard volunteering as a means to boost self-esteem, competence, and mood. Volunteering can also be perceived as a means to avoid personal problems since the service occupies the volunteers' time.

These five functions are beliefs about volunteerism. It can be argued that while prosocial value function is other-serving in nature, other functions are relatively more self-serving. Existing literature has not explored the relationship between different types of volunteering beliefs and purpose in life.

According to cognitive-motivational perspective, such as Ajzen[23], beliefs can result in favorable or unfavorable evaluative reactions manifested in cognition, feeling, motivation, or behavior[21]. Thus, stronger functional beliefs about volunteerism may lead to a stronger intention to volunteer[23]. Beliefs can also directly lead to a higher frequency of volunteering behavior. On the other hand, when we have an intention to do something, there is a tendency for us to actually perform the deed[23]. Intention acts as the bridge between beliefs and behavior. However, empirical research showed that the belief-intention, belief-behavior, and belief-intention-behavior links are not always stable[24]. There are many factors influencing these links, such as the specificity and clarity of beliefs and intention[23], task difficulty, and environmental influence.

Based on the assertions of the existential[2,8] and cognitive-motivational perspectives (e.g., influence of beliefs on intention and behavior), this study aims to explore the relationship among purpose in life, volunteering beliefs, intentions, and behavior. Against the above background, four research questions are addressed in this paper:

*Research Question 1: What is the relationship between purpose in life and beliefs about volunteerism?*

According to Frankl, purpose in life is the basic motive for human behavior that allows us to attain existential well-being. Based on the taxonomy of experiences of purpose in life by Reker and Wong[2], it was hypothesized that purpose in life would be positively correlated with prosocial beliefs about volunteerism (Hypothesis 1A). In other words, those with a higher level of purpose in life would have a stronger endorsement of the prosocial value function of volunteering. As beliefs can be classified as other-serving beliefs (prosocial value function) and self-serving beliefs (understanding function, socializing function, well-being function, and future plan function), it was also hypothesized that the relationship between purpose in life and prosocial beliefs would be stronger than that between purpose in life and self-serving beliefs (Hypothesis 1B). Finally, it was hypothesized that adolescents with a high level of purpose in life would display stronger prosocial beliefs about volunteerism than adolescents with a low level of purpose in life (Hypothesis 1C).

*Research Question 2: What is the relationship between purpose in life and volunteering intention?*

Based on the existential well-being literature[2,3], it was hypothesized that purpose in life would be positively associated with volunteering intention (Hypothesis 2A). It was also hypothesized that adolescents with a high level of purpose in life would display higher volunteering intention than adolescents with a low level of purpose in life (Hypothesis 2B).

*Research Question 3: What is the relationship between purpose in life and volunteering behavior?*

Based on the existential well-being literature, it was hypothesized that purpose in life would be positively associated with volunteering behavior (Hypothesis 3A). It was also hypothesized that adolescents with a high level of purpose in life would demonstrate higher involvement in volunteering behavior than those with a low level of purpose in life (Hypothesis 3B).

*Research Question 4: What are the inter-relationships among purpose in life, beliefs about volunteerism, volunteering intention, and behavior?*

Purpose in life was hypothesized to predict factors related to volunteerism. Ajzen formulated a model on the relationships among beliefs, intention, and behavior, where beliefs are antecedents of intention and intention is the antecedent of behavior[23]. In addition, beliefs can also be the direct antecedents of behavior[21]. In this study, a path model would be constructed to examine the relationships among purpose in life, volunteering beliefs, intention, and behavior. In particular, it was hypothesized that purpose in life would influence prosocial beliefs about volunteerism [2], which would subsequently affect volunteering behavior.

## METHOD

### Participants and Procedure

The data for the present analyses were derived from a large-scale cross-sectional survey on volunteer service patterns among Chinese adolescents in Hong Kong. A total of 5,946 secondary school (high school) students (2,193 boys [36.9%], 3,744 girls [63.1%], and nine respondents not indicating their gender) participated in the study. They were recruited from 31 secondary schools and one Protestant church by convenience sampling. Among the participants, 66% were junior-grade students (Secondary 1 to 3, with ages ranging from 13 to 15) and 34% were senior-grade students (Secondary 4 to 6, with ages ranging from 16 to 18). The mean age of the participants was 14.77 years (SD = 1.60).

Both parental and participant consents were obtained. Parental consent was sought by the researcher by sending letters to parents at the request of school authorities. For participant consent, students were asked if they did not want to participate in the study at the time of data collection administration (i.e., “passive” informed consent). All participants responded to all scales in the questionnaire and demographic characteristics in a self-administered format. Adequate time was provided for the participants with regard to completion of the questionnaire.

### Instruments

#### *Revised Personal Functions of Volunteerism Scale (RV)*

The Revised Personal Functions of Volunteerism Scale (RV) is a 20-item self-report measure of the participants’ perceived functions of volunteerism. The scale was modeled after the Volunteer Functions Inventory (VFI) by Clary et al.[22] and was developed with reference to the developmental characteristics of Chinese adolescents. There are research findings supporting the reliability and validity of this instrument[15].

The RV assesses the underlying beliefs about volunteerism in terms of functions using a 6-point scale ranging from 1 (strongly disagree) to 6 (strongly agree). Five functions were found, namely, (1) prosocial value function (PRO) (e.g., “I feel compassion towards people in need”), (2) understanding function (UN) (e.g., “Volunteering lets me learn things through direct, hands-on experience”), (3) socializing function (SO) (e.g., “I can meet friends in volunteer service”), (4) well-being function (WB) (e.g., “Volunteering increases my self-esteem”), and (5) future plan function (FU) (e.g., “Volunteering makes it easier for me to enter university or better schools”). The Cronbach’s $\alpha$ of the 20-item RV was 0.91. The reliabilities for five types of beliefs were 0.76 (prosocial), 0.87 (understanding), 0.79 (socializing), 0.86 (well-being), and 0.80 (future plan). Law showed that the scale scores were able to differentiate volunteers and nonvolunteers[15].

#### *Volunteering Intention Scale (VI)*

Based on literature[25], four items were developed to form the Volunteering Intention Scale (VI). The first item about the general volunteering intention used a 5-point scale ranging from 1 (no intention and with resistance) to 5 (extremely strong intention). The second item about whether one would pay attention to information about volunteer service used a 4-point scale ranging from 1 (never) to 4 (always). The third item about interest used a 6-point scale ranging from 1 (not interested with resistance) to 6 (extremely interested). The last item about the response to volunteering invitation also used a 6-point scale ranging from 1 (rejection and repulsion to inviter) to 6 (absolute participation). The score of each item was standardized by dividing the number of options, after which the average scores were then added. As there were four items, the highest score was 4. The Cronbach's α of the VI is 0.82. Law showed that the scale scores were able to differentiate volunteers and nonvolunteers[15].

#### *Service Hours*

Volunteering behavior was measured by self-reported total hours in serving the community within the past 12 months.

#### *Purpose in Life Scale (PIL)*

The Purpose in Life Scale (PIL) was designed by Crumbaugh and Maholick[26], and it was Shek[11] who validated the scale in the Chinese context. In this study, an abridged 7-item version was used. The reliability of the PIL was 0.87 in terms of Cronbach's α, with the PIL showing good internal consistency. Law showed that the scale scores were able to differentiate volunteers and nonvolunteers[15].

### RESULTS

#### Relationship between Purpose in Life and Beliefs on Volunteerism

Correlation coefficients on the relationship between purpose in life and beliefs on volunteerism are presented in Table 1. Two observations can be highlighted from the findings. First, purpose in life was significantly correlated with prosocial beliefs, thus supporting Hypothesis 1A. Second, except the understanding function, PIL scores were found to be strongly correlated with prosocial beliefs compared with other self-serving beliefs. When self-serving beliefs (understanding, well-being, socializing, and future plan functions) were grouped together, the correlation of purpose in life with other-serving beliefs was significantly higher than the correlation of purpose in life with self-serving beliefs ($z = 3.38$, $p < 0.05$) (Table 1). In short, the findings provided support for Hypothesis 1B.

Using the median PIL score (5.0) as the cutoff score, participants with "high" PIL (mean total score equal to 5 and above) and "low" PIL (mean total score lower or equal to 4.9) were identified. As shown in Table 2, adolescents with a high level of purpose in life displayed significantly stronger prosocial beliefs on volunteerism than adolescents with a low level of purpose in life, with a medium effect size (Cohen $d = 0.55$). The findings supported Hypothesis 1C.

#### Relationship between Purpose in Life and Volunteering Intention

The correlation coefficient between purpose in life and volunteering intention was significant with a moderate effect size ($r = 0.30$, $p < 0.001$). In addition, adolescents with a high level of purpose in life displayed higher volunteering intention than those with a low level of purpose in life, with a medium effect size (Cohen $d = 0.50$). These findings provided support for Hypotheses 2A and 2B.

**TABLE 1**
**Relationship between Purpose in Life and Beliefs on Volunteerism**

| Variable | PIL | Pairwise Comparison with PRO vs. PIL (*z* Score) |
|---|---|---|
| PRO | 0.33* | — |
| UN | 0.30* | 1.44 ns |
| WB | 0.21* | 6.98** |
| SO | 0.19* | 8.16** |
| FU | 0.07* | 14.58** |
| SELF | 0.27* | 3.38** |

PRO = prosocial value function; UN = understanding function; WB = well-being function; SO = socializing function; FU = future plan function; SELF (UN + WB + SO + FU) = self-serving beliefs (sum of understanding, well-being, socializing, and future plan scores).

*$p < 0.001$, **$p < 0.05$.

**TABLE 2**
**Comparison of Adolescents with High vs. Low Purpose in Life**

| Variable | High PIL | | Low PIL | | t Value | Cohen d |
|---|---|---|---|---|---|---|
| | Mean | SD | Mean | SD | | |
| PRO | 4.99 | 0.63 | 4.60 | 0.67 | –21.28* | 0.55 |
| VI | 2.55 | 0.59 | 2.25 | 0.61 | –19.02* | 0.50 |
| HR | 10.26 | 18.86 | 7.77 | 16.04 | –5.45* | 0.14 |

PRO = prosocial beliefs; VI = volunteering intention; HR = service hours.

*$p < 0.001$.

### Relationship between Purpose in Life and Volunteering Behavior

The correlation coefficient between purpose in life and volunteering behavior was significant, but showed a low effect size (r = 0.08, $p < 0.001$). Table 2 shows that adolescents with a high level of purpose in life spent more hours on volunteer service in the past 12 months than those with a low level of purpose in life, demonstrating a low effect size (Cohen d = 0.14). These findings provided support for Hypotheses 3A and 3B.

### Inter-Relationships among Purpose in Life, Beliefs on Volunteerism, Volunteering Intention, and Behavior

Standard multiple regression analyses on both behavior and intention were also performed. By using service hours as the dependent variable, results showed that while there were three significant predictors (intention, prosocial beliefs, and purpose in life), self-serving beliefs were not significant in the prediction of volunteering behavior (Table 3). On the other hand, all predictors of volunteering intention were significant (Table 4). Similar results controlling for gender and grades were found.

**TABLE 3**
**Standard Multiple Regression of Volunteering Intention, Beliefs, and Purpose in Life on Service Hours**

| Beta | | | | $R^2$ | $f^2$ |
|---|---|---|---|---|---|
| PIL | PRO | SELF | VI | | |
| 0.03** | 0.05** | 0.00 | 0.33* | 0.11 | 0.12 |

PIL = purpose in life; PRO=prosocial beliefs; SELF = self-serving beliefs; VI=volunteering intention.

*$p < 0.001$, **$p < 0.05$.

**TABLE 4**
**Standard Multiple Regression of Beliefs and Purpose in Life on Volunteering Intention**

| Beta | | | $R^2$ | $f^2$ |
|---|---|---|---|---|
| PIL | PRO | SELF | | |
| 0.14* | 0.21* | 0.33* | 0.29 | 0.41 |

PIL = purpose in life; PRO = prosocial beliefs; SELF = self-serving beliefs.

*$p < 0.001$.

A path model was constructed based on the findings presented in Tables 1, 3, and 4. The model is shown in Fig. 1. The total effect of purpose in life to service hours due to beliefs was the combination of the following: (1) the direct effect of purpose in life, (2) the indirect effect via belief, and (3) the indirect effect via belief and then via intention. The total effect of purpose in life due to self-serving beliefs was 0.06, whereas that due to prosocial beliefs was 0.07. The predictive ability of prosocial beliefs in this path model was marginally higher than self-serving beliefs.

### DISCUSSION

In view of the paucity of studies that examine the relationships between purpose in life and adolescent volunteering beliefs, intention, and behavior, the present study examined the following issues: (1) the relationship between purpose in life and different beliefs about volunteerism, (2) the relationship between purpose in life and volunteering intention, (3) the relationship between purpose in life and volunteering behavior, and (4) the inter-relationships among purpose in life and volunteering beliefs, intention, and behavior. Based on the thesis that purpose in life influences adolescent volunteering beliefs, intention, and behavior, several hypotheses were proposed.

Regarding Research Question 1, the findings showed that purpose in life is positively related to prosocial beliefs and that the correlation is stronger than those correlations based on self-serving beliefs. Furthermore, those experiencing a high level of purpose in life differed from those with a low level of purpose in terms of prosocial beliefs. There are two unique aspects of the related findings. First, the current

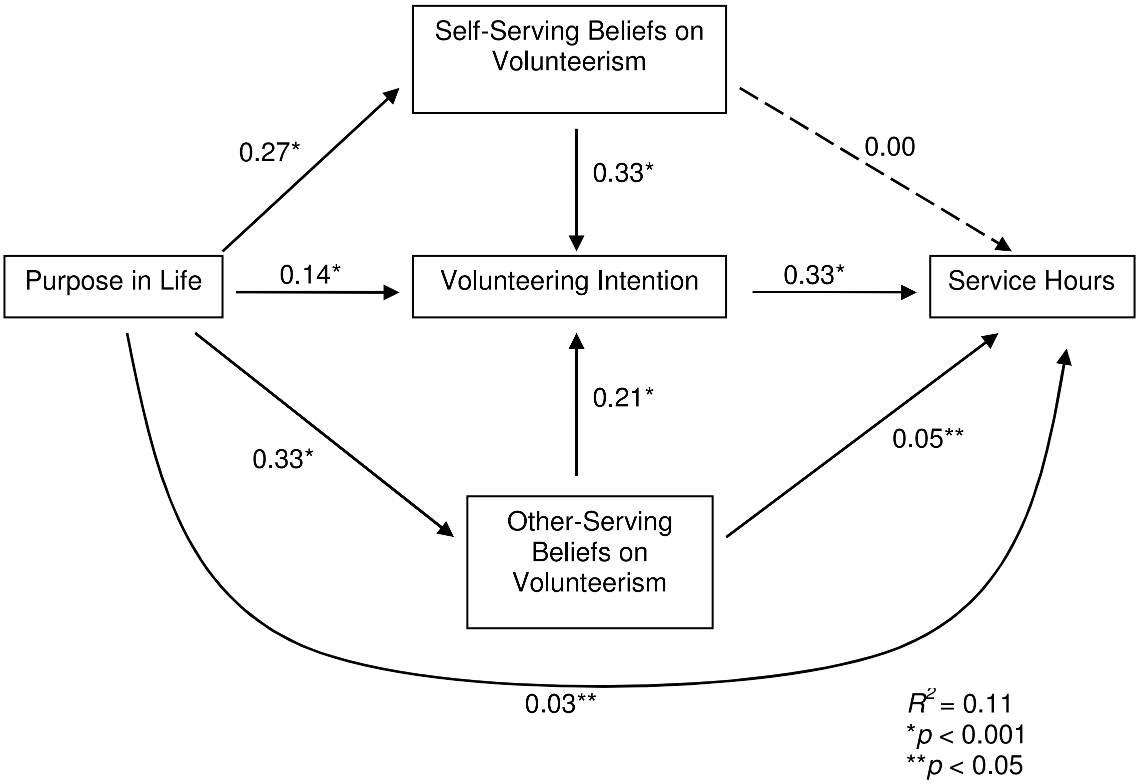

**FIGURE 1.** Path model showing inter-relationships among purpose in life, volunteering beliefs, intention, and behavior.

study is the first of its kind in its attempt to gain an understanding of the relationship between volunteering beliefs and purpose in life. It can thus be considered a groundbreaking study. Second, the present study clarified the relationship between purpose in life and beliefs about volunteerism. The present findings showed that different beliefs are, in varying degrees, related to purpose in life. Consistent with the assertion that prosocial value function belongs to a higher level of experience (Level 3 in Reker and Wong[2]), the present findings showed that the correlation between purpose in life and prosocial beliefs is significantly higher than the correlations between purpose in life and other self-serving beliefs (except understanding function).

Of course, one noticeably odd finding is that there seems to be no difference between the correlation between purpose in life and prosocial beliefs, and the correlation between purpose in life and understanding function (Table 1). The correlation between understanding function and prosocial value function is also high ($r = 0.61$, $p < 0.001$). In some sense, it can be argued that understanding function is not entirely self-serving. Hunter and Csikszentmihalyi[27] showed that adolescents with a higher level of learning attitudes tend to be concerned about people around them and the world (p. 33). Empathy and altruism were viewed as related. One element of empathy is perspective taking, which involves active understanding of people. Empathy induced by understanding social problems, especially social injustice, importance of human relationships, and worth of persons, is a prerequisite for active community participation[28]. Understanding function and prosocial value are thus related. One item of the RV is, in fact, related to understanding social problems ("I can learn more about social problems through volunteering"). The above arguments and evidence suggest that understanding may not be entirely a self-serving function.

The findings showed that there is a significant relationship between purpose in life and intention to volunteer, and that participants with "high" vs. "low" levels of purpose in life differed in their respective intentions to volunteer (i.e., Research Question 2). The current study is the first of its kind to investigate the relationship between volunteering intention and purpose in life. Similar to other studies[10,16], the present study also showed that purpose in life is related to volunteering behavior (i.e., Research Question 3). Similar to the findings of Shek, Ma, and Cheung[10], adolescents with a high level of purpose in life took part in more volunteering behavior than adolescents with a low level of purpose in life. The high correlation between purpose in life and intention/behavior can be explained by the existential perspective. Reker and Wong[2] suggested that people with a higher level of purpose in life devote more time to service and commitment to a larger social cause. The tendency to serve is stronger. This implies that not only one's behavior is affected by purpose in life; the corresponding intention is also affected. Given the lack of research between adolescent volunteerism and purpose in life, this is a significant theoretical contribution to existing literature.

Finally, the findings showed that purpose in life, volunteering beliefs, volunteering intention, and volunteering behavior were inter-related. The established belief-intention-behavior link concurred with the predictions of the cognitive-motivational perspective[21]. The path model suggests that other-serving beliefs can predict service hours directly, whereas self-serving beliefs must exert their influence on behavior through the mediation of volunteering intention. This highlights the importance of prosocial beliefs in the role of volunteering behavior. In addition, it should be noted that the correlation between purpose in life and volunteering behavior is only 0.08, while the variance explained is 0.006. The current model, which incorporates beliefs and intention, increases the prediction to 0.11. The present findings suggest that in addition to behavior, beliefs and intention are also essential in examining the relationship between volunteerism and purpose in life.

Several practical implications can be derived from the study. First, the present findings suggest that prosocial beliefs rather than self-serving beliefs should be emphasized in volunteer service training. Second, the means to enhance both beliefs and intention in an attempt to promote volunteering behavior could be devised. Third, the means to enhance purpose in life should be devised as well. In particular, positive youth development programs[29] should be developed to focus on purpose in life, prosocial beliefs on volunteerism, and volunteering intention. Volunteer services with adolescent volunteers should be arranged with the consideration of volunteering beliefs and intention.

There are several limitations of the present study. First, because of the cross-sectional nature of the study, the relationship between purpose in life and volunteerism can be bidirectional in nature. As such, longitudinal studies[18] can gain more insights into the understanding of the relationship. Second, an abridged 7-item version of the Purpose in Life Scale instead of the 20-item full form was used in this study. The scale in full form consists of two factors, namely, "purpose of existence" and "quality of existence"[11]. Obviously, a more detailed understanding can be derived from the unabridged version of the form. Third, as the study adopted a large sample with 5,946 participants chosen through convenience sampling, the generalizability of the current findings should be examined with caution. Despite the limitations, with reference to the comment of Thoits and Hewitt[18] that the relationship between personal well-being (quality of life) and volunteering "has not often been examined in the literature" (p. 117), the present study can be regarded as a constructive response to the literature on quality of life.

### ACKNOWLEDGMENTS

This paper is based on the Ph.D. thesis of the first author under the supervision of the second author. The preparation for this paper was financially supported by Wofoo Foundation Limited.
